# Antidepression of Xingpijieyu formula targets gut microbiota derived from depressive disorder

**DOI:** 10.1111/cns.14049

**Published:** 2022-12-22

**Authors:** Yannan Li, Lixuan Yang, Junnan Li, Wei Gao, Zhonghui Zhao, Kaiqiang Dong, Wenzhe Duan, Baoan Dai, Rongjuan Guo

**Affiliations:** ^1^ Second Clinical Medical College Beijing University of Chinese Medicine Beijing China; ^2^ Department of Neurology Dongfang Hospital Beijing University of Chinese Medicine Beijing China; ^3^ Department of Mental Health Tsinghua University Yuquan Hospital Beijing China

**Keywords:** astrocyte, depressive disorder, glycometabolism, gut microbiota, tradition Chinse medicine

## Abstract

**Objective:**

This investigation aims to determine the antidepressant role of Xingpijieyu formula (XPJYF) mediated via gut microbiota (GM)–brain axis.

**Methods:**

We collected fecal microbiota from patients with depressive disorder (DD) and cultured microbiota in vitro. Some of microbiota were transplanted into germ‐free rats with the intragastric administration of XPJYF grain at the dose of 1.533 g/kg/day. The behaviors were studied by forced swimming test, open field test, sucrose preference test, and body weight. Products of hypothalamus–pituitary–adrenocortical (HPA) axis, neurotransmitter, and serum cytokines were investigated by enzyme linked immunosorbent assay. Glial fibrillary acidic protein (GFAP), a biomarker of astrocyte, was quantified using immunofluorescence. Microbiota culturing in vitro after XPJYF treatment was analyze by 16 s RNA sequencing technology. We used lipopolysaccharide (LPS) to mimic activated rat primary astrocyte in vitro. Brain‐derived neurotrophic factor (BDNF), cytokines, and oxidative stress factors were determined by western blotting, and glycometabolism in astrocyte was investigated by 2‐deoxy‐D‐glucose (2‐DG) uptake, adenosine triphosphate (ATP), and glucose‐1‐phosphate (G1P) kits.

**Results:**

Microbiota composition during 8 mg/ml of XPJYF (H12‐8) for 12 h showed the more consistency. *Lactococcus* is enriched in DD‐derived microbiota composition, and *Biffdobacterium and Lactobacillus* in H12‐8 group. GLUCOSE1PMETAB‐PWY and PWY‐7328 of which biofunctions were dominantly encoded by *Biffdobacterium* were the top two of altered pathways. XPJYF improved behaviors and repressed astrocyte activation in depression rats. XPJYF elevated 2‐DG uptake, ATP, glucose‐1‐phosphate, and brain‐derived neurotrophic factor (BDNF), and inhibited cytokines and oxidative stress in LPS‐induced astrocyte.

**Conclusion:**

XPJYF treatment targets inflammation, activation, and glycometabolim in astrocyte via gut microbiota modulation, thereby improve animal behaviors, HPA axis dysfunction, and neurotransmitter synthesis in depression rats.

## Graphical abstract

On the one hand, XPJYF treatment inhibits astrocyte activation resulting in the reduction of cytokines that play key roles in depression‐related neuroinflammation; on other hands, XPJYF promotes glycometabolism in astrocyte via triggering Bifidobacteium enrichment, ameliorating neuronal injury in depression.

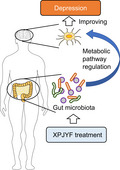

## 1 INTRODUCTION

Globally, depressive disorder (DD) is the most common mental health illness and the fourth leading disability.^1^ It is estimated that the lifetime prevalence of DD ranges from 2% to 21%.^2^ DD is heterogeneous and diverse resulting in a poor understanding of pathophysiology, thereby impeding the development of therapeutic strategies. Patients with depression develop the pathological alteration in central nervous system (CNS). The brain region controling the complex emotion is significanlty altered with the neuronal atrophy. Particularly, the altered neuronal strcture in prefrontal lobe is one of pathological basics since it impairs the communication between neurons.^3^, ^4^, ^5^ Importantly, neuroinflammation‐inolved immune disorder contributes to the alteration in the specific brain region. For example, Belge et al showed the volume of certain brain regions was increased with the reduction of proinflammatory cytokines.^6^ Also, inflammatory response was elevated in the prefrontal lobe from patients with depression, suggesting neuroinflammation may contribute to the altered prefrontal lobe during depression. Moreover, inflammation may evoke the elevated synthesis of histamine leading to the decrease of serotonin, a neurotransmitter involved in DD pathogenesis.^7^, ^8^ Therefore, depression‐related neuroinflammation is associated with the alteration in prefrontal lobe. Neuroinflammation is mainly triggered by glia cells, such as astrocyte mediating the release of proinflammatory cytokines after stimulated extracellular stimulation.^9^ Thus, astrocyte may play the key role of inflammation in prefrontal lobe during depressive disorder.

Gut microbiota (GM) can contact with CNS to maintain the physiological framework.^10^ During the occurrence and development of disease, GM can interact with CNS to modulate cell death via inflammation mapping from gut to brain. Interestingly, investigations focusing on gut microbiota in depression have been increased. GM has been determined to involve the function and condition in the brain. A report recruiting patients with cognitive impairment concluded GM was significantly correlated with local brain spontaneous activity and cognitive function.^11^ GM‐derived metabolites function as the key role in driving the activity and development of the brain.^12^ Further, GM can indirectly or directly communicate with CNS via modulating cytokines from immune cell, inducing hypothalamus–pituitary–adrenocortical (HPA) axis, and releasing metabolites to brain based on circulation.^13^ In other words, GM defined as the virtual organ contributing to the homeostasis and health interacts with CNS via neuroendocrine and metabolic pathways.^1^ Metabolites links GM and inflammation response in neurological disorders. Mechanistically, GM alteration may stimulate the change of phenotype in glia cells via neuroendocrine and metabolic pathways, thereby induce neuroinflammation and depressive‐like behaviors. Jiang et al.^14^ reported that GM from rats with alcohol‐induced DD could activate microglia and promote neuroinflammation. For astrocyte, GM treatment has been found to improve alteration in astrocyte under chronic unpredictable stress.^15^ The GM‐to‐astrocyte hypothesis provides the potential of therapeutic prospect based on targeting microbiota modulation to astrocyte‐involved neuroinflammation. For instance, Lv et al.^16^ reported that the therapeutic mechanism of melatonin was depended on GM‐mediated short‐chain fatty acid that was related to activated glia (astrocyte and microglia) and subsequent neuroinflammation in depression. Therefore, GM may contribute to the modulation of astrocytic neuroinflammation during depression progression. GM mechanism provides the novel insight into antidepressant drug investigations.

Chinese herbal medicine has the potential of long‐term antidepression strategy due to slow onset and mild efficacy. Given the oral administration of Chinese herbal medicine (CHM), we assume that CHM potentially improves depressive symptom via the GM–brain axis. An experimental study^17^ unraveled Kaixinsan, a compound of CHM, could restore microbiota composition in gut, thus ameliorating the inflammation in the brain and depressive‐like behaviors in mice with chronic unpredictable mild stress, suggesting the potential of CHM in treating GM‐mediated depression. In traditional Chinese medicine, depressive disorder (DD) is defined as the concept characterized by the symptom of liver depression and spleen deficiency (sLDSD). Xingpijieyu formula (XPJYF) is a compound of CHM, consisting of *Panax quinquefolius*, *Acorus tatarinowii*, *Curcuma longa L*, and *Hypericum perforatum L*. XPJYF is found to play an anti‐depressive role in rats stimulated by chronic unpredictable mild stress via restoring mitochondrial function.^18^ Intragastric administration of XPJYF indicates the question that XPJYF improves depressive‐like behaviors via GM‐mediated neuroinflammation.

In this investigation, we collect fecal microbiota from sLDSD and explore the characteristics of microbiota culturing in vitro and in vivo after XPJYF treatment based on 16 s RNA sequencing technology. Also, microbiota is transplanted into germ‐free rats to determine the GM–brain axis‐mediated antidepression effect of XPJYF based on the animal model with fecal microbiota transplantation. Meanwhile, we plan to explore the mechanism of XPJYF in depression‐induced astrocyte activation via LPS‐stimulated astrocyte.

## 2 METHODS

### 2.1 In vitro culturing technology

The samples collected from patients with sLDSD were divided in two parts. One part was used for microbiota culturing in vitro. In anaerobic conditions at 37°C, the fecal samples were homogenized in anaerobic PBS (100 mg in 1 ml PBS), and subsequently divided in control, H12‐1, H12‐4, H12‐8, H24‐1, H24‐4, and H24‐8 groups. After serial dilution, all groups were seeded onto Petri dishes supplemented with YCFA medium containing 2 mg/ml of glucose, maltose, and cellobiose. In control group, microbiota was cultured in YCFA medium without XPJYF treatment. In H12‐1, H12‐4, and H12‐8 groups, XPJYF was supplemented in YCFA medium at 1, 4, and 8 mg/ml for 12‐h incubation, respectively. In H24‐1, H24‐4, and H24‐8 groups, microbiota was incubated with 1, 4, and 8 mg/ml XPJYF in YCFA medium for 24 h, respectively. After incubation, microbiota samples were harvested for 16 s RNA sequencing to visualize the microbiota community.

### 2.2 Fecal microbiota transplantation (FMT)

Another part of fecal samples was used for FMT in germ‐free rats. Before FMT, 6‐weeks‐old germ‐free rats were purchased from Institute of Experimental Animals, the Chinese Academy of Medical Sciences (Beijing, China). In the aseptic flexible‐plastic isolator, recipient animals were housed one per cage at 20 ± 1°C with 12 h of light/dark cycle in 59 ± 1% humidity. Each recipient could freely access to the autoclaved feed and water. Recipient rats were randomly divided into four groups: control group (*n* = 8) without FMT, Con‐microbiota group (CC_mic) (*n* = 8) exposed to fecal microbiota from healthy individuals, DD‐microbiota group (DD_mic) (*n* = 8) exposed to depression microbiota, and XPJYF group (*n* = 8) based on the administration of DD‐microbiota group and treated with XPJYF after a 2‐weeks FMT. Fresh fecal pellets were collected from DD patients and healthy individuals and processed to form a slurry microbiota solution. Recipient animals were given 2 ml of microbiota solution by gavage once a day for 2 weeks. These recipients then were treated with XPJYF grain by intragastric administration for 2 weeks at the dose of 1.533 g/kg/day. Body weight of rats was investigated once a week. All animal experiments and protocols have been reviewed and approved by the Animal Care and Use Committee of Lab Animal Research Institute of China Academy of Medical Science, and the approval number was ZH22001.

### 2.3 Behavior tests

For forced swimming test (FST), each rat was placed in a 20‐cm‐diameter and 50‐cm‐height glass cylinder containing the 35‐cm‐deep water where the rat forcibly swam for 6 min at 24°C ± 1°C. The time of immobility was measured in 6 min (2 min for pre‐test and 4 min for test) to describe depression. For open field test (OPT), each rat was placed into a plastic without‐top chamber (72 × 72 × 40 cm^3^) which was cleaned using 70% ethanol before tests. The rat could freely move for 5 min around the chamber and a digital camera recorded standing times of the hind limb as horizontal activity score. For sugar preference assay, each rat in the cage could access to two bottles of 1% sucrose water for 1‐day of acclimatization, and then one of sucrose water was replaced by a bottle of pure water for the 1‐day of adaptation. The formal experiment started after 24 h of fasting and drinking. A 1%‐sucrose‐water‐containing bottle and a bottle supplemented with pure water were placed in the cage, and the weight of the two bottles were recorded as S1 and P1, respectively. After a 24‐h test, both bottles were removed from each cage for weighting. The rest weight of the two bottles were recorded as S2 and P2, respectively. The sugar preference was calculated as follows:

$$\text{Sugar preference}=\frac{S1-S2}{\left(S1+P1\right)-\left(S2+P2\right)}\times 100\%.$$

### 2.4 Enzyme linked immunosorbent assay for Neurotransmitter, HPA axis, and Cytokines

After the last behavior tests, rats were euthanized by excessive anesthesia. We collected the peripheral blood samples from the abdomen and separated the prefrontal lobe. After peripheral blood standing for 30 min or more, serum samples were acquired. Prefrontal lobe samples were homogenated in normal saline (1:9, w/v), followed by the centrifugation for 10 min at 4°C and 5000 *g*. ELISA was used for the measures of tumor necrosis factor‐α (TNF‐α), interleukin‐6 (IL‐6), interleukin‐1 (IL‐1) and interferon‐γ (IFN‐γ), corticosterone (CORT), adrenocorticotropic hormone (ACTH), and corticotropin releasing hormone (CRH) in serum, and 5‐hydroxytryptamine (5‐HT), noradrenaline (NE), dopamine (DA) in prefrontal lobe. TNF‐α (RTA00), IL‐6 (R600B), IL‐1 (RLB00), IFN‐γ (RIF00) kits were purchased from R&D Co. Lt. (Minnesota, USA); 5‐HT (RA20506), NE (RA20557), and DA (RA20050) kits from Bioswamp Co.Lt. (Wuhan, Hubei Province, China); and CORT (E‐EL‐0160c), ACTH (E‐EL‐R0048c), CRH (E‐EL‐R0270c) from Elabscience Biotechnology Co.Lt. (Wuhan, Hubei Province, China).

### 2.5 Immunocytochemistry for glial fibrillary acidic protein (GFAP)

For prefrontal lobe sections, these sections were performed by dewaxing, hydration, and antigen repair before immunocytochemistry. Astrocyte with different treatment were seeded into 96‐well plates for immunocytochemistry. Samples were incubated in a 4% paraformaldehyde solution overnight at 4°C, followed by blocking with 5% normal goat serum (Solarbio, Beijing, China) for 1 h at room temperature and cultured using the anti‐GFAP antibody (ab7260, Abcam, Cambridge, UK) at 4°C overnight. An anti‐Rabbit IgG (Abcam, 1/300) was added to the sections, and the samples were incubated overnight at 4°C in the dark before re‐staining of 594‐conjugated AffiniPure Donkey (Jackson, USA, 1/1000) for 7–8 min at room temperature. The fluorescence intensity was measured using confocal laser scanning microscope (Olympus, Japan).

### 2.6 Cell culture

Rat primary cortical astrocytes were purchased from Gibco (USA). Astrocyte cultured in T25 flasks were incubated with DMEM (high glucose) containing 15% fetal bovine serum at 37°C in 5% CO_2_, and the medium was changed every 4 day. For cellular experiment, cells were seeded into 12‐well plates.

### 2.7 Glycose metabolism test

Astrocyte were seeded into 12‐well plates and divided into three groups: control group without LPS stimulation and XPJYF treatment, LPS group stimulated by 1 mg/ml LPS, and XJPYF group treated with 8 mg/ml XPJYF for 12 h based on LPS stimulation. After different treatment, cells were collected to investigate the role of XPJYF in glycose metabolism in LPS‐induced astrocyte based on 22‐DG uptake, glucose‐1‐phosphate (G1P) level and adenosine triphosphate (ATP) content that were measured by Glucose Uptake Assay Kit (Ab136955, Abcam, Shanghai, China), Glucose‐1‐phosphate assay kit (Ab155892, Abcam, Shanghai, China), ATP Assay kit (s0026, Beyotime, Shanghai, China).

### 2.8 Western blotting

Cellular protein was extracted from astrocyte using RIPA lysis buffer (Beyotime, Shanghai, China), followed by the separation with sodium dodecyl sulfate (SDS)– polyacrylamide gel electrophoresis (PAGE). Protein was transplanted onto polyvinylidene difluoride (PVDF) membrane (Millipore, USA) and blocked with 5% skimmed milk for 90 min at room temperature. Membranes were incubated with anti‐TNF‐α (1/1000, 17590‐1‐AP, Proteintech, Wuhan, China), interleukin‐1β (IL‐1β) (1/1000, 12242s, Cell Signaling Technology, USA), IL‐6 (1/1000, 21865‐1‐AP, Proteintech), cyclooxygenase‐2 (COX‐2) (1/1000, 12375‐1‐AP, Proteintech), inducible nitric oxide synthase (iNOS) (1/1000, 18985‐1‐AP, Proteintech), and BDNF (1/1000, 28205‐1‐AP, Proteintech) overnight at 4°C, respectively. After that, membranes were cultured with secondary antibody for 60 min at 37°C, followed by the visualization using ECL kit. Glyceraldehyde‐3‐phosphate dehydrogenase (GAPDH) (1/10,000, 60004‐1‐g, Proteintech) was used for protein control. Integrated density of blot image was measured by Image J software. The relative expression of protein was calculated as follows: the relative expression = integrated density of test protein/integrated density of GAPDH expression.

### 2.9 16s DNA sequencing

Total bacterial DNA was extracted via TIANamp Bacteria DNA kit (Tiangen, Beijing, China), followed by the 16s RNA sequencing technology manipulated by Personalbio Compony (Shanghai, China). High throughput sequencing was a procedure in Illumine Novaseq (Illumine, USA) via paired‐end method. The V3–V4 region of 16 S RNA was amplified using polymerase chain reaction (PCR) with forward primer (F: 5′‐ACT CCT ACG GGA GGC AGC A‐3′) and reverse primer (R: 5′‐GGA CTA CHV GGG TWT CTA AT‐3′). 16 s RNA gene reads were aligned in Greengenes database.

### 2.10 Microbiome analysis

In our study, QIIME 22019.4 was used for microbiome bioinformatics. Raw sequences were demultiplexed using the demux plugin followed by primers cutting with cutadapt plugin. The sequences were subsequently merged, filtered, and dereplicated in Vsearch. The sequences were clustered to format operational taxonomic units (OTUs). Genes on species taxonomy was annotated according to Greengenes database. To obtain the relative abundance of microbiota, Qiime feature‐table rarefy was used for the rarefaction of OTUs. Chao1 (for species richness), Simpson (for species diversity), and Shannon (for species diversity) were calculated to describe the α‐diversity of gut microbiota via ggplot2 package. To determine the alteration in β‐diversity, Bray‐curtis distance was calculated for dimensionality reduction via principal coordinate analysis (PCoA) using ape package. Linear discriminant analysis coupled with effect size measurements (LEfSe) was used to determine the biomarker of each group. Phylogenetic investigation of communities through reconstruction of unobserved states (PICRUSt) tool was used to investigate the metabolic function of altered gut microbiota. The enrichment analysis of metabolism pathways was performed according to Kyoto Encyclopedia of Genes and Genomes (KEGG) database via metagenomeSeq package. Hierarchical clustering was performed by ggtree package based on unweighted pair‐group method with arithmetic means.

### 2.11 Statistical analysis

Data from animal experiments were expressed as mean ± standard error of mean (SEM), and these from cellular experiments shown as mean ± standard deviation (SD), followed by the drawing of GraphPad 8.0. Statistical analysis was investigated using SPSS 22.0 (IBM, USA). Shapiro–Wilk test was used for normality test. The difference in data that exhibited a normal distribution was analyzed by one‐way analysis of variance, followed by Dunnett's multiple comparison test. The difference was identified when *p* value was <0.05 at 95% confidence interval.

## 3 RESULT

### 3.1 Characterization of microbiota composition based on OTUs

**FIGURE 1:**
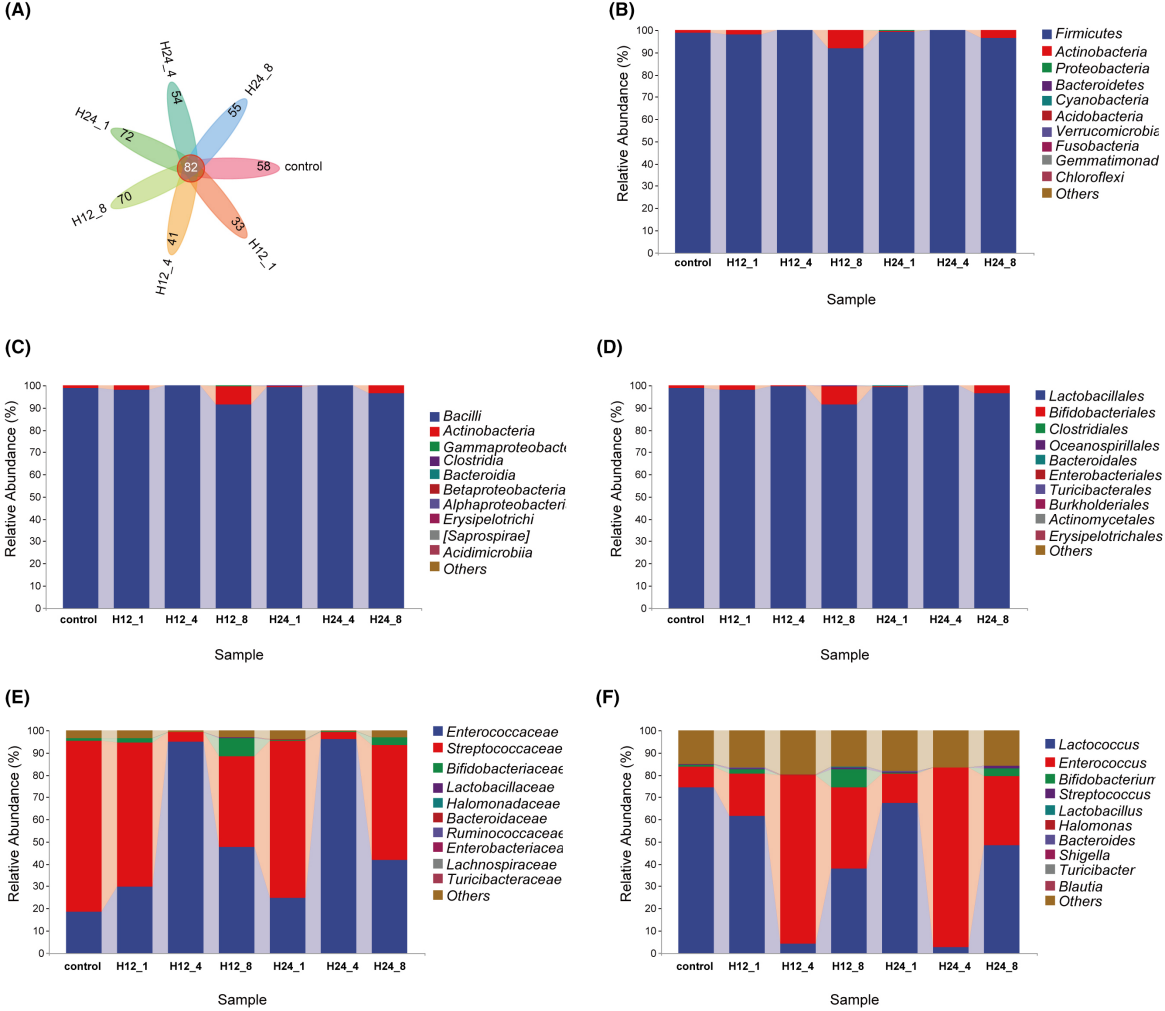
Characterization of microbiota composition based on OTUs. (A) OTUs. (B) Microbiota composition at the phylum level. (C) Microbiota composition at the class level. (D) Microbiota composition at the order level. (E) Microbiota composition at the family level. (F) Microbiota composition at the genus level.

Compared with Control group (OTUs = 58), OTUs of H12‐1 and H12‐4 groups decreased to 33 and 41, respectively, and nevertheless, that of H12‐8 group increased to 70 (Figure 1A). OTUs of H24‐1, H24‐4, and H24‐8 groups were 72, 54, and 55, respectively. We concluded the changes of OTUs in H12‐8 and H24‐1 groups. As shown in Figure 1B–D, at the phylum, class and order level, the dominant bacteria were *Fimicutes*, *Bacilli* and *Lactorbacacillales*. At the family level, *Enterococcaceae* was the most dominant, followed by *Streptococcaceae* and *Bifidobacteriaceae* (Figure 1E). Also, *Lactococcus*, *Enterococcus* and *Bifidobacterium* were the top three of relative abundance at the genus level (Figure 1F). Collectively, there was the obvious change in microbiota composition due to XPJYF treatments at the different doses.

### 3.2 The analysis of altered microbiota based on α, β‐diversity

**FIGURE 2:**
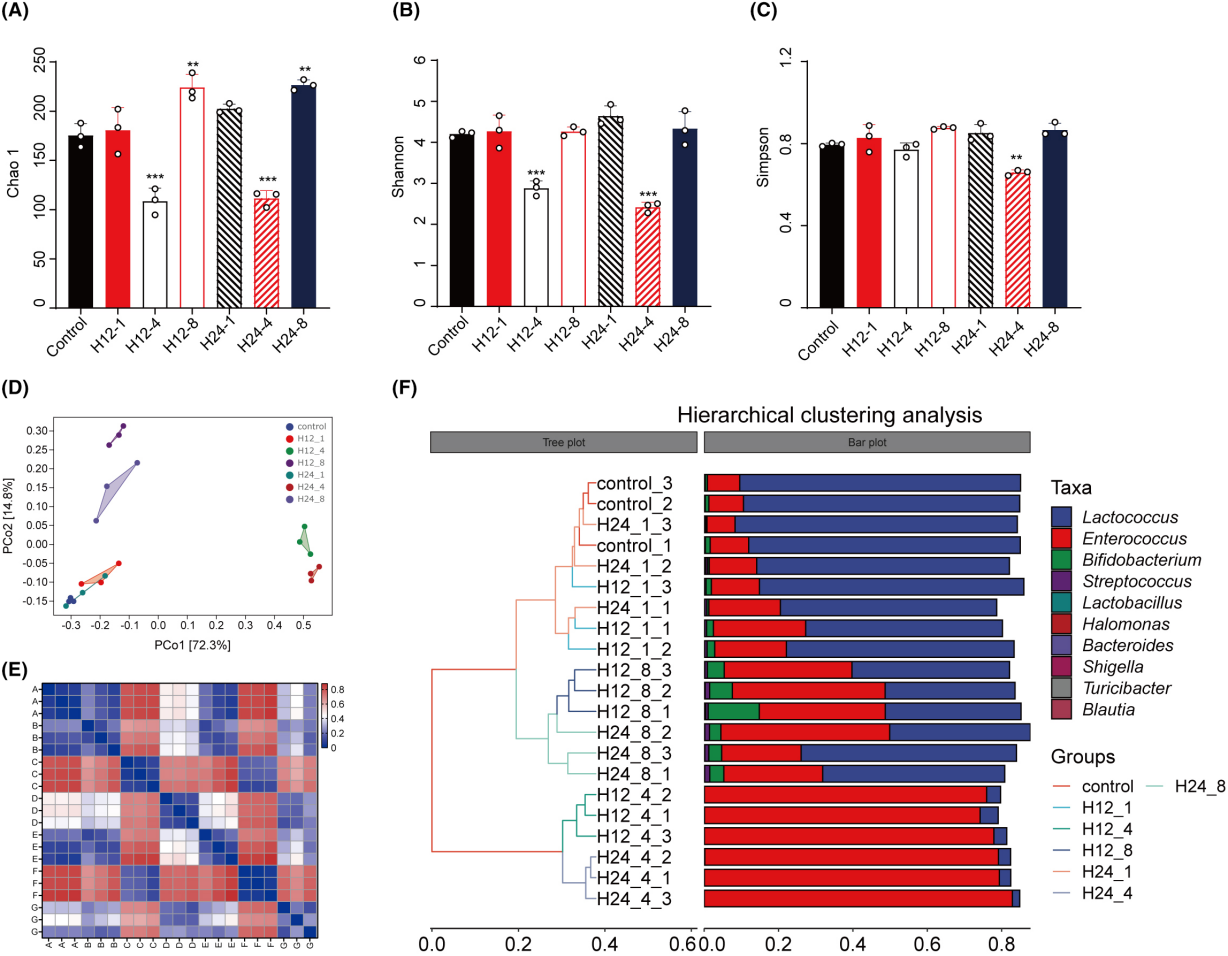
The analysis of altered microbiota based on α, β‐diversity. (A) Chao1. (B) Shannon. (C) Simpson. (D) PCoA analysis based on Bray Curtis distances. (E) Bray Curtis distances. (F) Hierarchical clustering analysis based on Bray Curtis distances.

For the Chao1 index, there were the significant reductions in H12‐4 and H24‐4 groups and the elevations in H12‐8 and H24‐8 groups (Figure 2A). For the Shannon index, we found only these of H12‐4 and H24‐4 groups obviously decreased compared with control group (Figure 2B). Also, the Simpson index of H24‐4 group showed the only significant alteration (Figure 2C). PCoA analysis based on Bray Curtis distances showed the marked alteration of bacteria composition among the seven group (Figure 2D). Moreover, hierarchical clustering analysis based on Bray Curtis distances revealed the more consistency in H12‐8 group (Figure 2E,F).

### 3.3 The analysis of microbiota biomarkers and functional prediction based on LEfSe and PICRUSt

**FIGURE 3:**
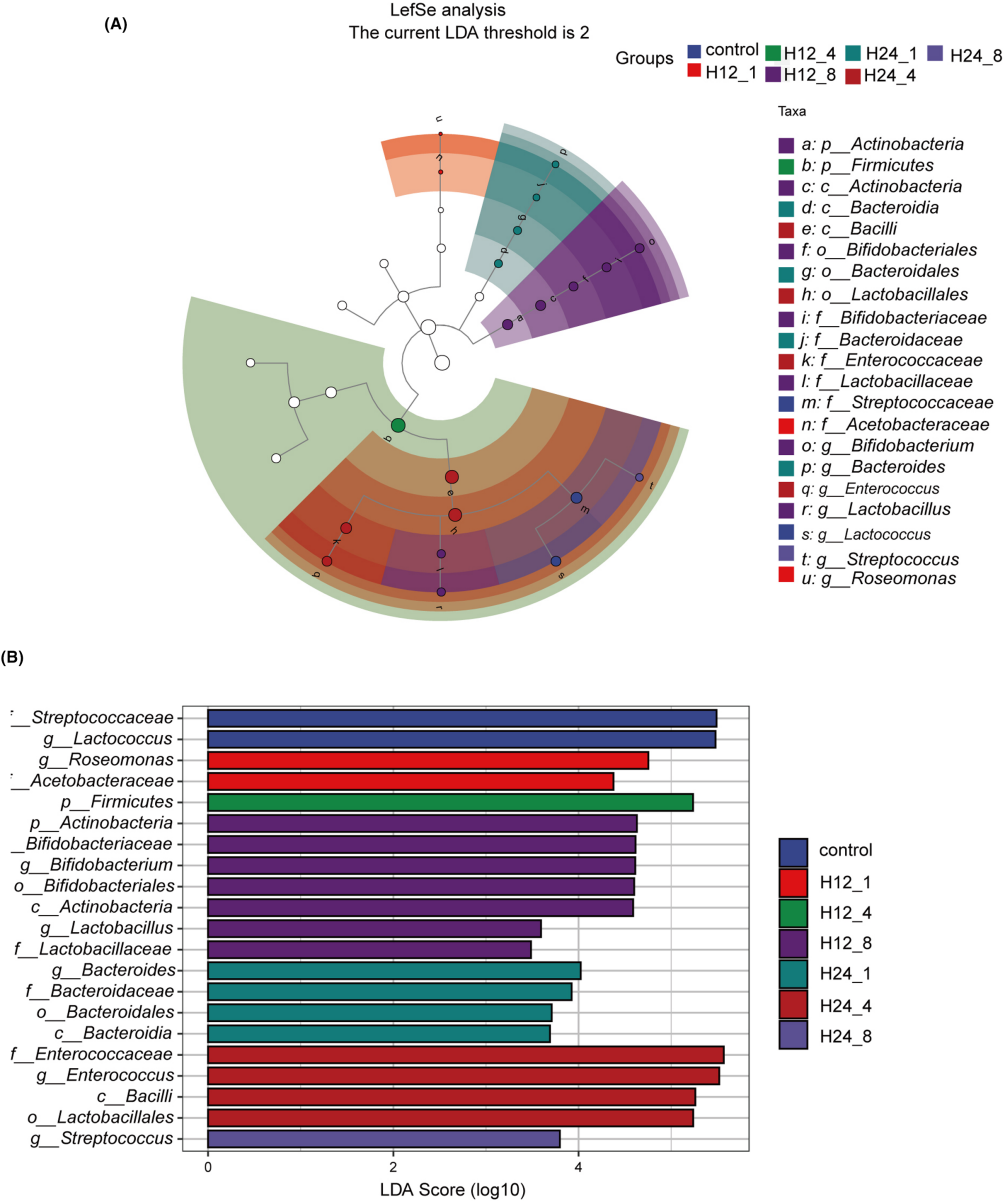
LEfSe analysis of altered microbiota among seven groups. (A) Cladogram with taxonomic hierarchical distribution of biomarkers in each group. (B) Histogram based on LDA value showing biomarkers in each group.

**FIGURE 4:**
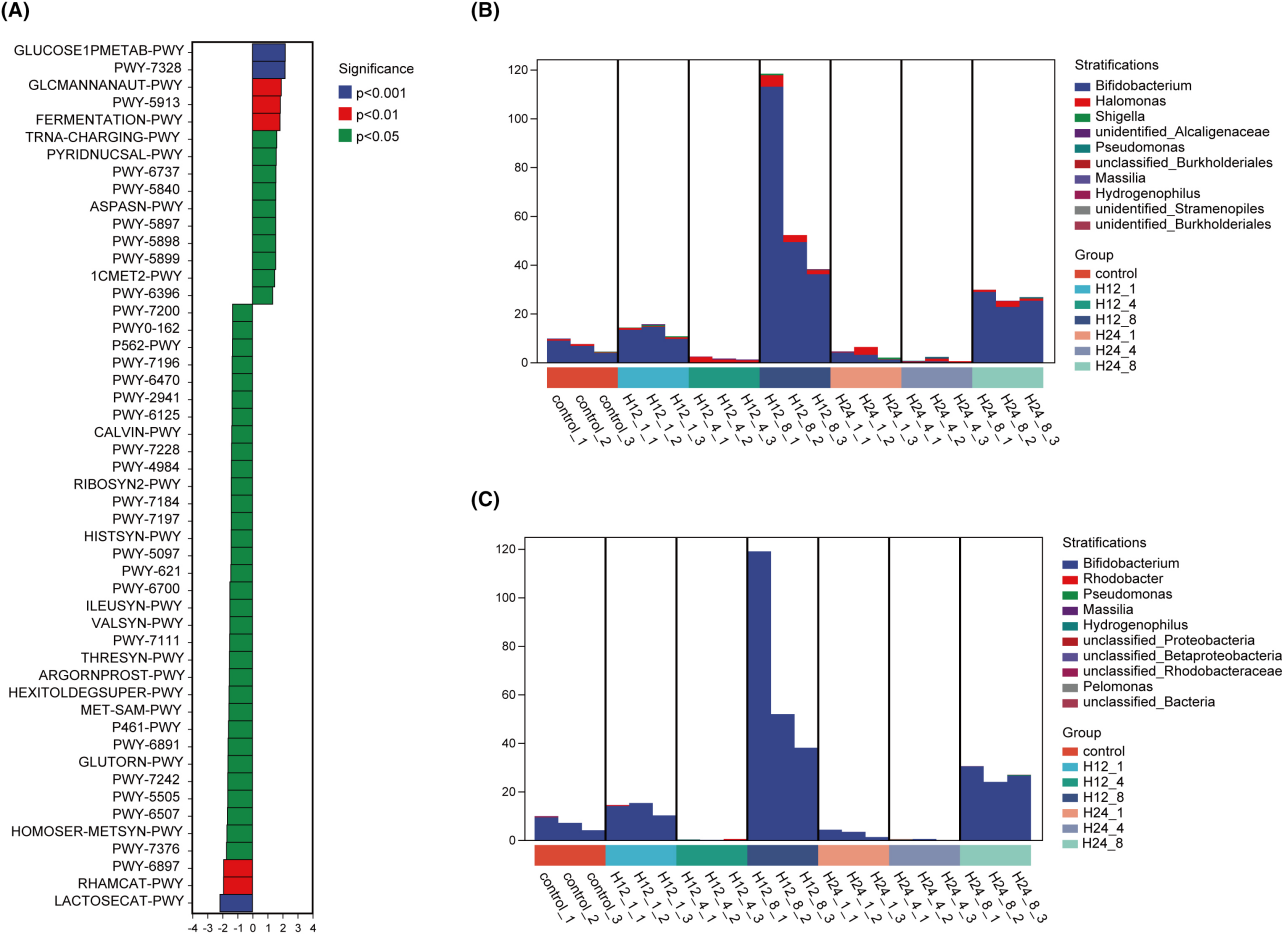
The functional prediction of altered microbiota based on PICRUSt. (A) Significantly altered pathway after XPJYF treatment. (B) Composition of genus associated with GLUCOSE1PMETAB‐PWY. (C) Composition of genus involved in PWY‐7328.

Firstly, we analyzed the biomarkers of each group based on LEfSe method (Figure 3). As shown in Figure 3, *Lactococcus*, *Roseomonas*, *Bacteroides*, *Enterococcus*, and *Streptococcus* were predominant genera in control, H12‐1, H24‐1, H24‐4, and H24‐8 groups, respectively. Two genera including *Biffdobacterium* and *Lactobacillus* were enriched in H12‐8 group. We inferred *Lactococcus* genus might be the biomarker of DD. Then, the function prediction of enriched microbiota was process by PICRUSt. After XPJYF treatment, 15 of pathways were identified as the upregulated pathways whereas 35 of pathways significantly were downregulated (Figure 4A). Particularly, there were the top two of altered pathways including glucose and glucose‐1‐phosphate degradation (GLUCOSE1PMETAB‐PWY) and superpathway of UDP‐glucose‐derived O‐antigen building blocks biosynthesis (PWY‐7328). Ulteriorly, we explore the dominant microbiota encoding the biofunction of GLUCOSE1PMETAB‐PWY and PWY‐7328 at the genus level. Intriguingly, the genus of *Biffdobacterium* (Figure 4B,C) was the largest contributor in manipulating the two biological pathway.

### 3.4 XPJYF treatment improved depressive rats transplanted with DD‐derived fecal microbiota

**FIGURE 5:**
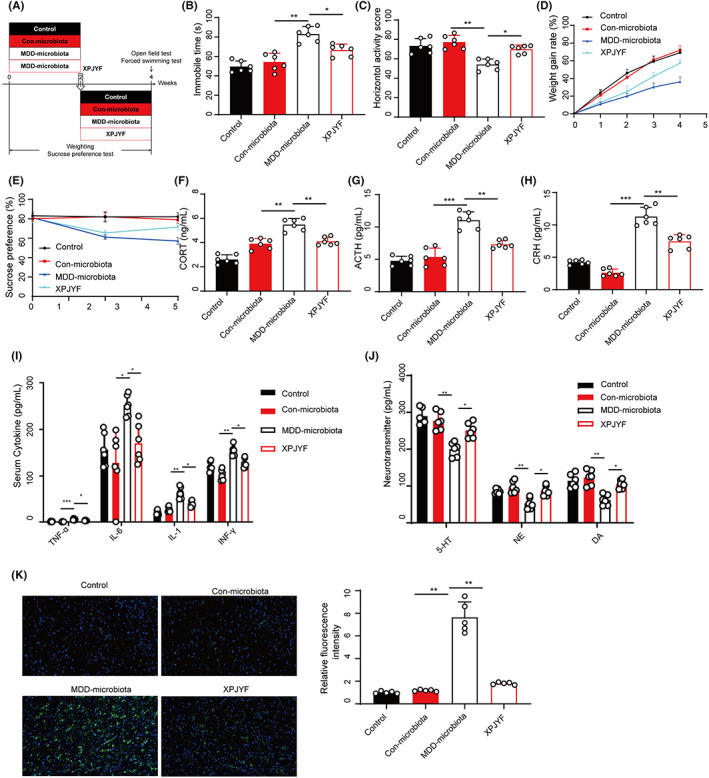
XPJYF treatment improved depressive rats transplanted with sLDSD‐DD‐derived fecal microbiota. (A) Experimental timeline of FMT from healthy individuals and patients with sLDSD‐DD. (B) Immobile time of FST. (C) Crossing counts of OFT. (D) gain rate of body weight. (E) Sugar preference. (F) CORT level. (G) ACTH level. (H) CRH level. (I) Levels of TNF‐α, IL‐6, IL‐1, and IFN‐γ in serum. (J) Levels of 5‐HT, NE and DA. (K) Astrocyte activation‐based GFAP.

Based on the role of XPJYF treatment in DD‐derived microbiota in vitro, we established the animal model to determine whether XPJYF treatment improved the depressive‐like symptom due to DD‐derived fecal microbiota in vivo. We isolated and cultured gut microbiota from patients with DD and subsequently the microbiota was transplanted into recipient animals followed by the XPJYF treatment (Figure 5A). We found microbiota from DD significantly extended immobile time of FST (Figure 5B) and reduced crossing counts (Figure 5C), body weight (Figure 5D), and sugar preference (Figure 5E). Hormone disorder in HPA axis was involved in the pathogenesis of depressive disorder. As shown in Figure 5F–H, DD‐derived microbiota markedly increased the levels of CORT, ACTH, and CRH, indicating the significant depressive symptom in recipient animals. Also, DD‐derived microbiota statistically enhanced cytokines (TNF‐α, IL‐6, IL‐1, and IFN‐γ) in serum of recipients and suppressed neurotransmitter including 5‐HT, NE, and DA (Figure 5I,J). Importantly, XPJYF treatment reversed these alterations in animals transplanted with DD‐derived microbiota showing the potential of ameliorating depressive symptom. Moreover, we explored the changes in gut microbiota of the animals. XPJYF treatment significantly altered gut microbiota composition of rats transplanted with DD‐derived microbiota (Figure S1A–D). Importantly, XPJYF decreased Firmicutes/Bacteroidetes ratio of DD‐derived microbiota in vivo (Figure S1B). Also, α‐ (Figure S1E–G) and β‐diversity (Figure S1H) in gut microbiota in rats with DD‐derived microbiota were affected by XPJYF treatment. These microbiological results showed the significant regulator role of XPJYF treatment in gut microbiota in vivo. Interestingly, XPJYF treatment repressed DD microbiota‐elevated astrocyte activation in prefrontal lobe (Figure 5K), revealing that therapeutic mechanism of XPJYF might target astrocyte activation in depressive disorder.

### 3.5 XPJYF treatment targeted astrocyte activation and glucose metabolism

**FIGURE 6:**
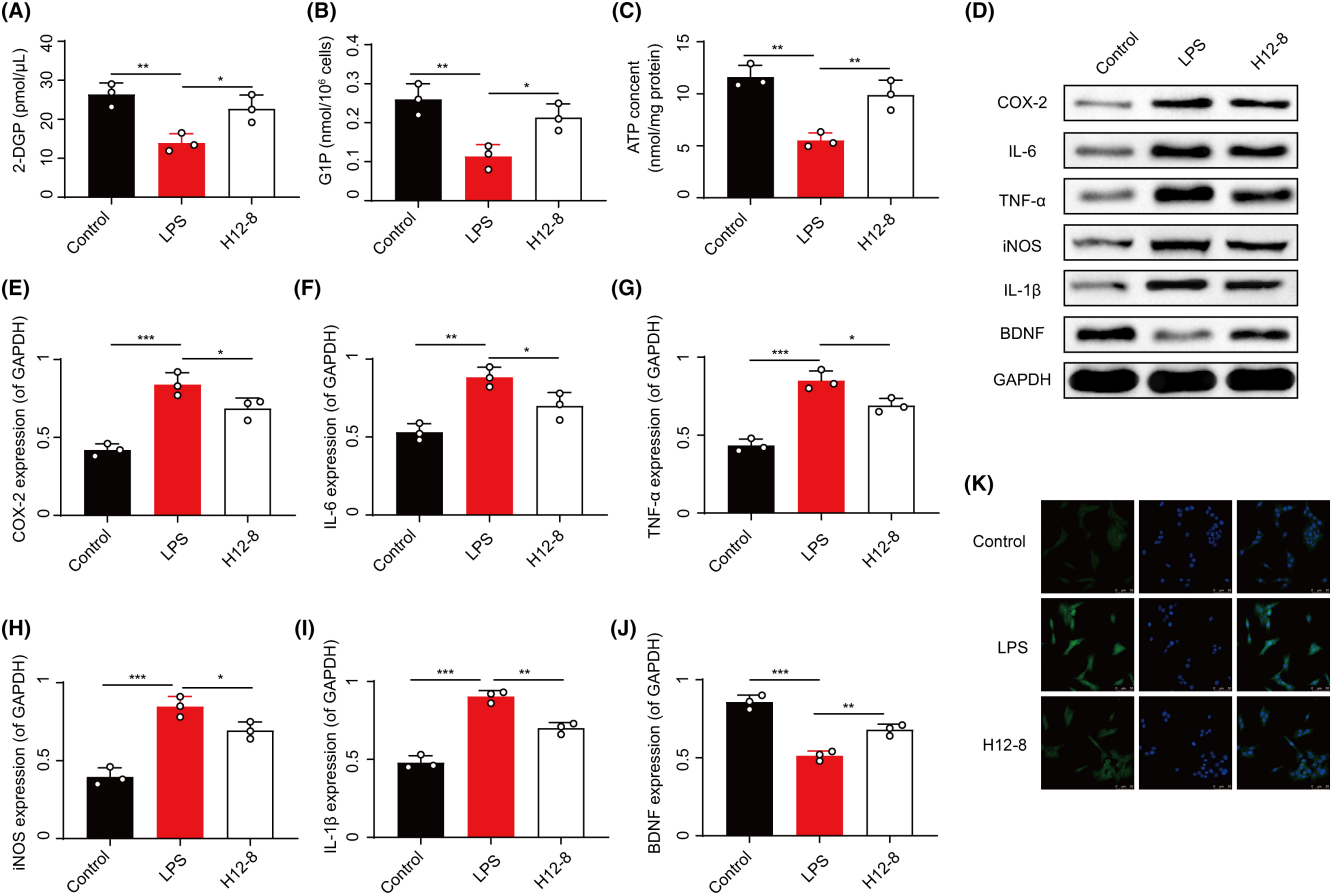
XPJYF treatment targeted astrocyte activation and glucose metabolism. (A) Uptake of 2DGP in astrocyte. (B) Glucose‐1‐phosphate level in astrocyte. (C) ATP content. (D) Western blotting. (E) The relative expression of COX‐2 in astrocyte. (F) IL‐6 expression in astrocyte. (G) TNF‐α expression in astrocyte. (H) iNOS expression in astrocyte. (I) IL‐1 expression in astrocyte. (J) BDNF expression in astrocyte. (K) Astrocyte activation by GFAP immunofluorescence.

Given XPJYF treatment inhibited astrocyte activation in animals with depression, we established LPS‐induced astrocyte to investigate the potential mechanism of XPJYF in activated astrocyte. As the abovementioned results in 3.3, *Biffdobacterium* enriched in H12‐8 group encoded the biofunction of GLUCOSE1PMETAB‐PWY and PWY‐7328 that were associated with glucose metabolism. Therefore, we estimated the glucose metabolism in LPS‐induced astrocyte based on glucose uptake, glucose‐1‐phosphate level, and ATP content. Surprisingly, XPJYF treatment elevated LPS‐inhibited uptake of 2DGP in astrocyte as well as glucose‐1‐phosphate and ATP showing that this treatment participated in glucose and energy metabolism during astrocyte activation (Figure 6A–C). Activated astrocyte was characterized by inflammation and oxidative stress. Thus, we next determined the inflammation and oxidative stress in XPJYF‐treated astrocyte. According to Figure 6D–I, XPJYF treatment significantly repressed LPS‐enhanced expressions of COX‐2, IL‐6, TNF‐α, iNOS, and IL‐1 in activated astrocyte. BDNF was mainly produced from astrocyte whose elevation contributed to the improvement of depressive disorder. Finally, we demonstrated that XPJYF treatment promoted BDNF production (Figure 6J) and inhibited fluorescence intensity of GFAP (Figure 6K) against the inhibition from LPS stimulation in astrocyte. Collectively, we conjectured that XPJYF treatment promoted BDNF expression and inhibited astrocyte activation via glucose metabolism, inflammation, and oxidative stress.

## 4 DISCUSSION

We show here that gut microbiota (GM) is characterized by a specifical microbial composition that the genus *Lactococcus* is enriched in response to depressive disorder (DD). Our results reveal exposed to XPJYF induces composition alteration of GM in vitro, thereby reshaping the activation and glycometabolism of astrocyte. These findings suggest the anti‐depression of XPJYF treatment in DD linking gut microenvironment and astrocyte in brain via microbiota‐associated metabolism.

In tradition Chinese medicine, DD is the symptom of liver depression and spleen deficiency (sLDSD). With no serious side effects, treatment with Chinese herbal medicine (CHM) plays the significant anti‐depressive role in depressive disorder.^19^ A previous report^20^ determines that DD‐derived microbiota (depression microbiota) induces significant depressive‐like disorder in recipient rats via mitochondrion‐associated neuronal metabolism and immune in brain. According to this finding, we further investigate the anti‐depression role of XPJYF in rats stimulated by depression microbiota. The occurrence and development of depressive disorder revolves around elevation in inflammation, imbalance of neurotransmitter, and dysregulation in HPA axis.^21^, ^22^, ^23^ We determine XPJYF treatment can ameliorate depressive‐like behaviors in depression microbiota‐stimulated rats based on FST, OPT, and sugar preference assay. In depressive‐like rats, we found XPFJY could decrease the immobile time of FST, and increase sucrose preference and the horizontal activity score of OPT, indicating the antidepressant role of XPJYF. Meanwhile, XPJYF treatment promotes neurotransmitter synthesis and alleviates the disorder of inflammation and HPA axis. XPJYF compound has been proved to improve the capability of spatial learning and memory in chronic unpredictable stress‐induced depressive disorder in the investigation by Wang et al.^24^ Our findings accord with this investigation. Particularly, we find XPJYF treatment inhibited depression microbiota‐induced astrocyte activation based on the enhancement of GFAP, a biomarker of astrocyte. Steinacker et al.^25^ concluded that GFAP was positively associated with depression severity, showing astrocyte activation might be the marked event during depression progression. Also, Michel et al.^26^ showed unipolar depression was characterized by GFAP upregulation in cerebrospinal fluid. However, GFAP in prefrontal cortex is found to be decreased in depressive disorder. These findings suggest that there is the reginal specificity of astrocyte activation. At present, we hold the view that astrocyte disorder represents pathological alteration in brain during depression. Moreover, we notice that XPJYF treatment represses cytokines and oxidative stress in activated astrocyte and promotes BDNF expression. In CNS, astrocyte can stimulate cytokines and oxidative stress to evoke neuroinflammation that increase susceptibility to depression. Increasing reports show anti‐depression drugs elevate BDNF expression in resistance to neuroinflammation‐induced depression.^27^ BDNF is mainly expressed in astrocytes. Therefore, XPJYF treatment possibly regulated BDNF expression in astrocyte to function as the anti‐inflammation and anti‐depression effects. Given XPJYF‐induced alteration of proteins in astrocyte, we demonstrate astrocyte may be the therapeutic target of XPJYF treatment in recipients with depression microbiota. Notably, XPJYF treatment altered gut microbiota environment of rats with DD‐derived microbiota, suggesting XPJYF might target gut microbiota to regulate depressive symptom.

The pathological condition induces intestinal barrier impairment that contributes to the altered GM composition, which cause microbiota‐derived metabolites such as LPS entering the brain to trigger neuroinflammation based on the circulatory system.^28^ In the pathogenesis of depressive disorder, GM–brain axis acts as the distinctive role, triggering inflammation in gut and next infiltrating in both periphery and central nervous system, with the altered functions of glia such as microglia and astrocyte.^29^, ^30^ Mechanistically, gut microbiota produces metabolites into microenvironment leading to phenotype transformation of immune cells, alteration in blood–brain barrier, and modulation for glia cells, thus evoking neuroinflammation‐associated depression progression.^31^ *Lactococcus* is one of probiotic that induces GM modulation to restore neurotransmitters synthesis, epigenetic effects, and HPA axis‐involved stress response in CNS.^32^, ^33^ *Lactococcus* has a potential role in mitigating depressive‐like behaviors.^34^ However, our results suggest that *Lactococcus* is enriched in DD patients, indicating this genus can be the biomarker to distinguish depressive symptom. Seemingly, *Lactococcus* enrichment in DD is contrary to its biofunction in depressive‐like behaviors. We infer that its enrichment may be the early acute response to depression‐induced gut dysbiosis, therefore functioning as the initial anti‐depression to repress depressive‐like behaviors.^34^, ^35^ In other words, *Lactococcus* enrichment may be the early marked event of DD. At the early stage, *Lactococcus* is rapidly increased to resists the DD‐induced pathophysiological alteration via producing beneficial substances. However, detrimental bacteria are dominant with the development of DD, thereby offsetting the improvement of *Lactococcus*.

Microbiota‐based antidepressant drugs are increasingly prominent focusing on antimicrobial mechanism in depression. Mounting evidence suggests that supplement with traditional Chinese medicine regulates metabolites in brain via GM modulation, which causes the reduction of depressive‐like behaviors.^36^, ^37^ Considering the improvement role of XPJYF treatment in depression microbiota‐induced depressive symptom, we assume that there is a potential mechanism of XPJYF treatment in gut microbiota modulation. Our findings show that microbiota composition is different due to the different doses of XPJYF treatment. Treatment with 8 mg/ml of XPJYF for 12 h shows interesting influence in GM from DD based on OTUs, PCoA, and Clustering. Ulteriorly, we conclude this therapeutic scheme triggers probiotic enrichment (*Bifidobacteium* and *Lactobacillus*) according to LEfSe analysis. Interestingly, there is the most significant upregulation of two glucose‐associated pathways after XPJYF treatment, of which biofunction is majorly encoded by *Bifidobacterium*. *Bifidobacterium* is the critical component in GM to maintain organism healthy.^38^ These results imply that XPJYF treatment possibly modulates glycometabolism in depressive disorder via targeting *Bifidobacteium*‐involved metabolites that develops the decrease of depression‐induced behaviors and physiological alterations. Also, we observe XPJYF treatment promotes glycometabolism in activated astrocyte. Glycogen turnover in astrocyte is the key mechanism maintaining normal mood.^39^ We conclude that XPJYF treatment enhances glucose uptake and glucose‐1‐phosphate that is a precursor for glycogen synthesis, and a production for glycogen breakdown, thereby reversing astrocyte injury caused by glycogen decrease and, in turn, improving depressive disorder.^40^ The elevation of glucose‐1‐phosphate may be attributed to the recovery of glycomtabolism. Astrocyte can be affected by gut microbiota. Given the biofunction of *Bifidobacteium* in glycometabolism, we provide a hypothesis that glycometabolism improvement in astrocyte is attributed to *Bifidobacteium* encoding glucose‐associated pathways, which subsequently decreases neuronal vulnerability and susceptibility to depression,^41^ leading to the improvement of depressive‐like behaviors in recipient mice. It still needs a follow‐by study to further verify the relationship between *Bifdobacteium* and astrocyte glycometabolism based on the animal model transplanted with *Bifidobacteium*.

Unfortunately, we failed to explore the function map of *Lactobacillus*, another biomarker in H12‐8 group. The mixture of *Lactobacillus* and *Bifidobacteium* can repress NF‐κB, TNF‐α, and bacterial lipopolysaccharide in depression,^42^ showing that there is the potential synergistic effect between the two microbiotas. Thus, *Lactobacillus* may cooperate with *Bifidobacteium* to be involved in glycometabolism and activation in depression‐induced astrocyte, or other depressive pathophysiological alteration. It needs further studies to verify the hypothesis.

We determine the antidepressant role of XPJYF treatment via gut–brain axis. XPJYF treatment inhibits astrocyte activation resulting in the reduction of cytokines that are key roles in depression‐related neuroinflammation; furthermore, XPJYF treatment promotes glycose metabolism in astrocyte via triggering *Bifidobacteium* enrichment, ameliorating neuronal injury in depression. Also, we infer *Lactococcus* enrichment may be the marked event that contributes to the early diagnosis of depressive disorder.

## AUTHOR CONTRIBUTIONS

Yannan Li contributed to concepts, design, experimental studies, writing‐original draft preparation. Lixuan Yang contributed to literature research. Junnan Li contributed to data analysis. Wei Gao did data analysis. Zhonghui Zhao did data acquisition. Kaiqiang Dong did statistical analysis. Wenzhe Duan did experimental studies. Baoan Dai did statistical analysis. Rongjuan Guo did supervision, writing‐reviewing, and editing. All the authors approved for the final version.

## FUNDING INFORMATION

This work was supported by National Natural Science Foundation of China (81874422, U21A20401).

## CONFLICT OF INTEREST

The authors declare that they have no conflict of interest.

## Supporting information


Figure S1.

Figure S2.

## Data Availability

The datasets used or analyzed during the current study are available from the corresponding author on reasonable request.
