# Supplementary figures and images for: Antidepression of Xingpijieyu formula targets gut microbiota derived from depressive disorder
Source: CNS Neurosci Ther. 2022 Dec 22;29(2):669–81. doi: 10.1111/cns.14049 (PMC9873506; doi:10.1111/cns.14049)

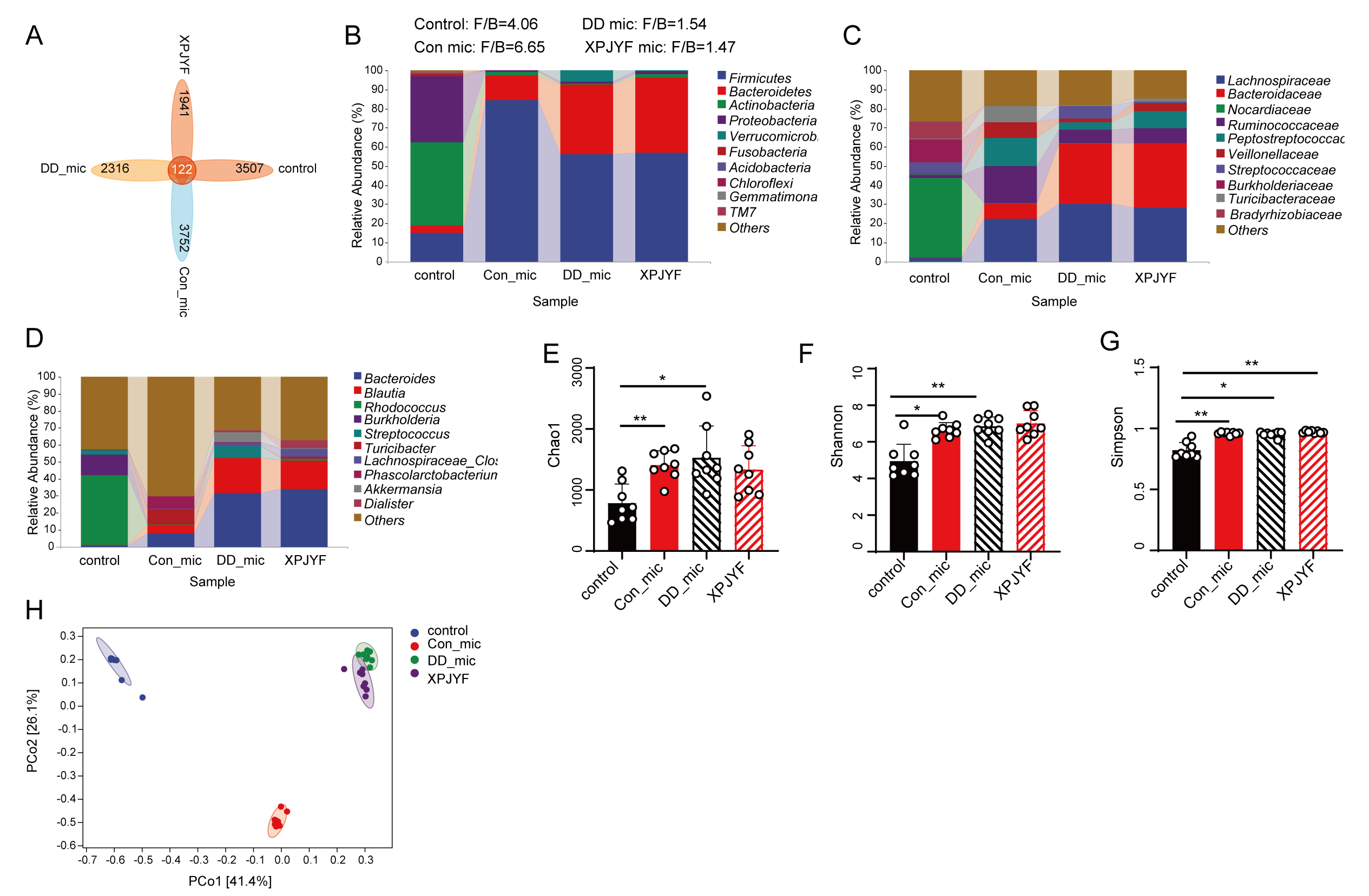

Supplement: Supplementary file 1 — Figure S1. [file CNS-29-669-s002.jpg]
