# Supplementary material for: Antidepression of Xingpijieyu formula targets gut microbiota derived from depressive disorder
Source: CNS Neurosci Ther. 2022 Dec 22;29(2):669–81. doi: 10.1111/cns.14049 (PMC9873506; doi:10.1111/cns.14049)
Supplement: Supplementary file 2 — Figure S2. [file CNS-29-669-s001.pdf]

Full unedited gel/blot of BDNF for Figure 6D

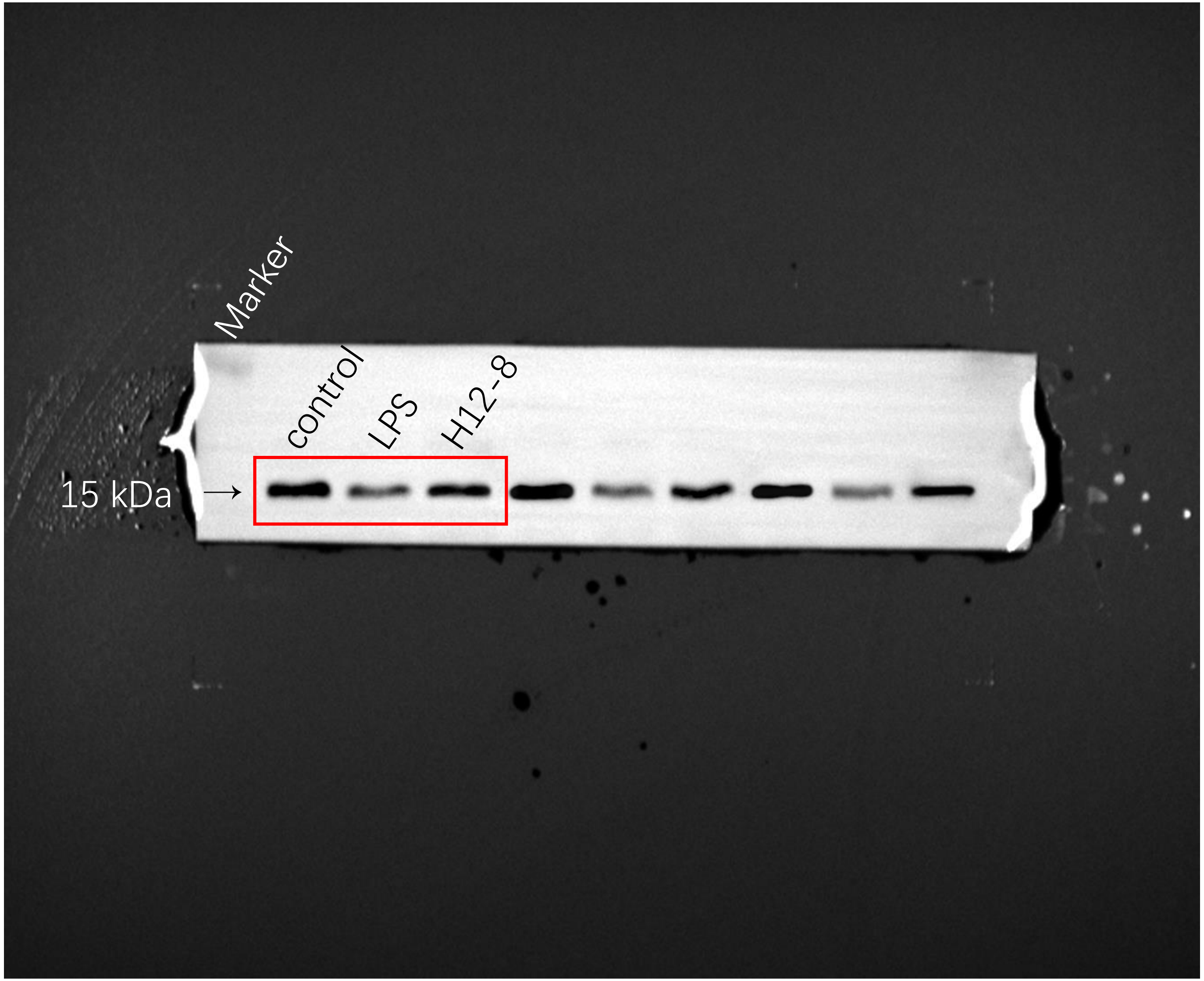


the cropped image in the manuscript were highlighted in red

Full unedited gel/blot of COX-2 for Figure 6D

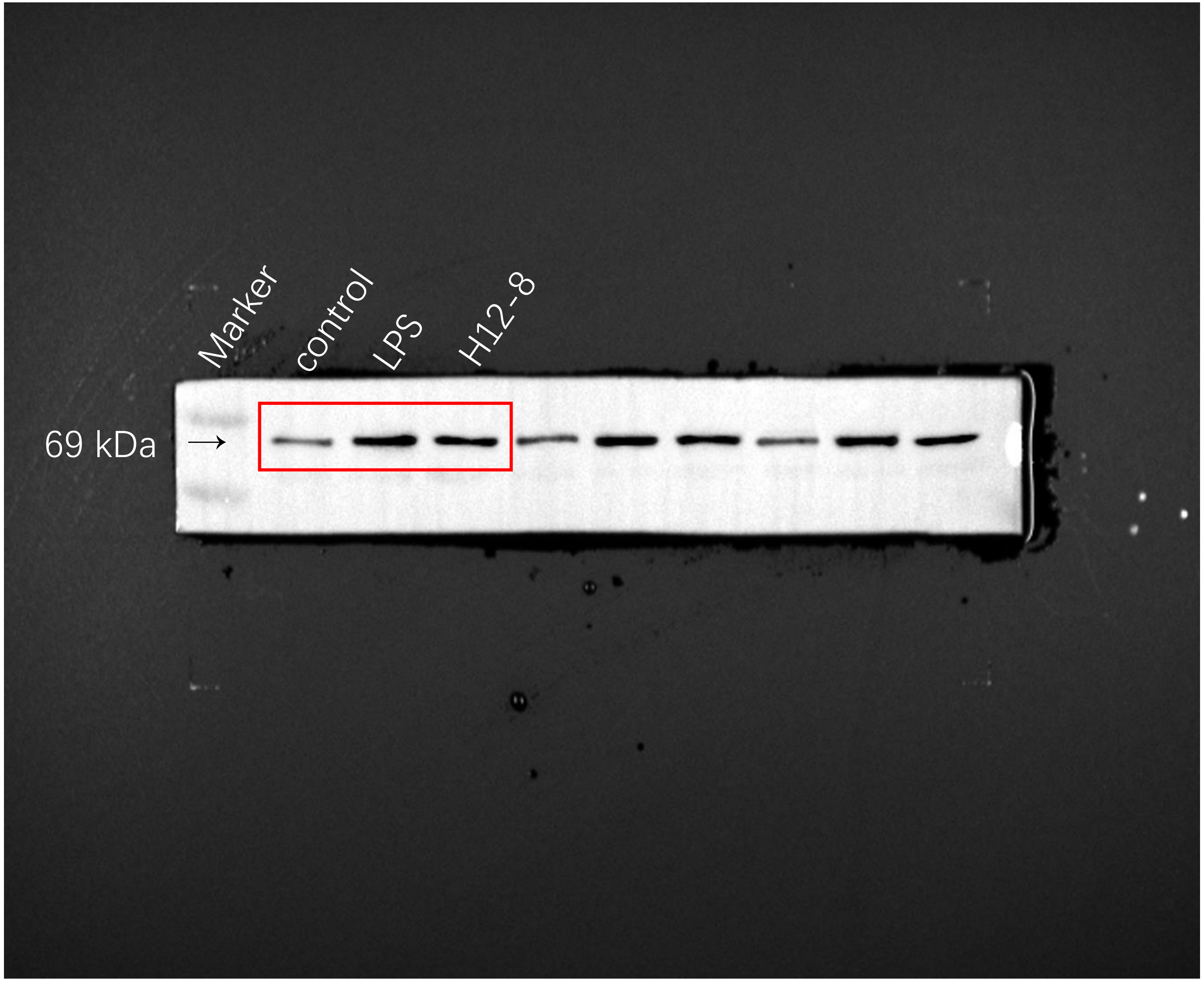


the cropped image in the manuscript were highlighted in red

Full unedited gel/blot of GAPDH for Figure 6D

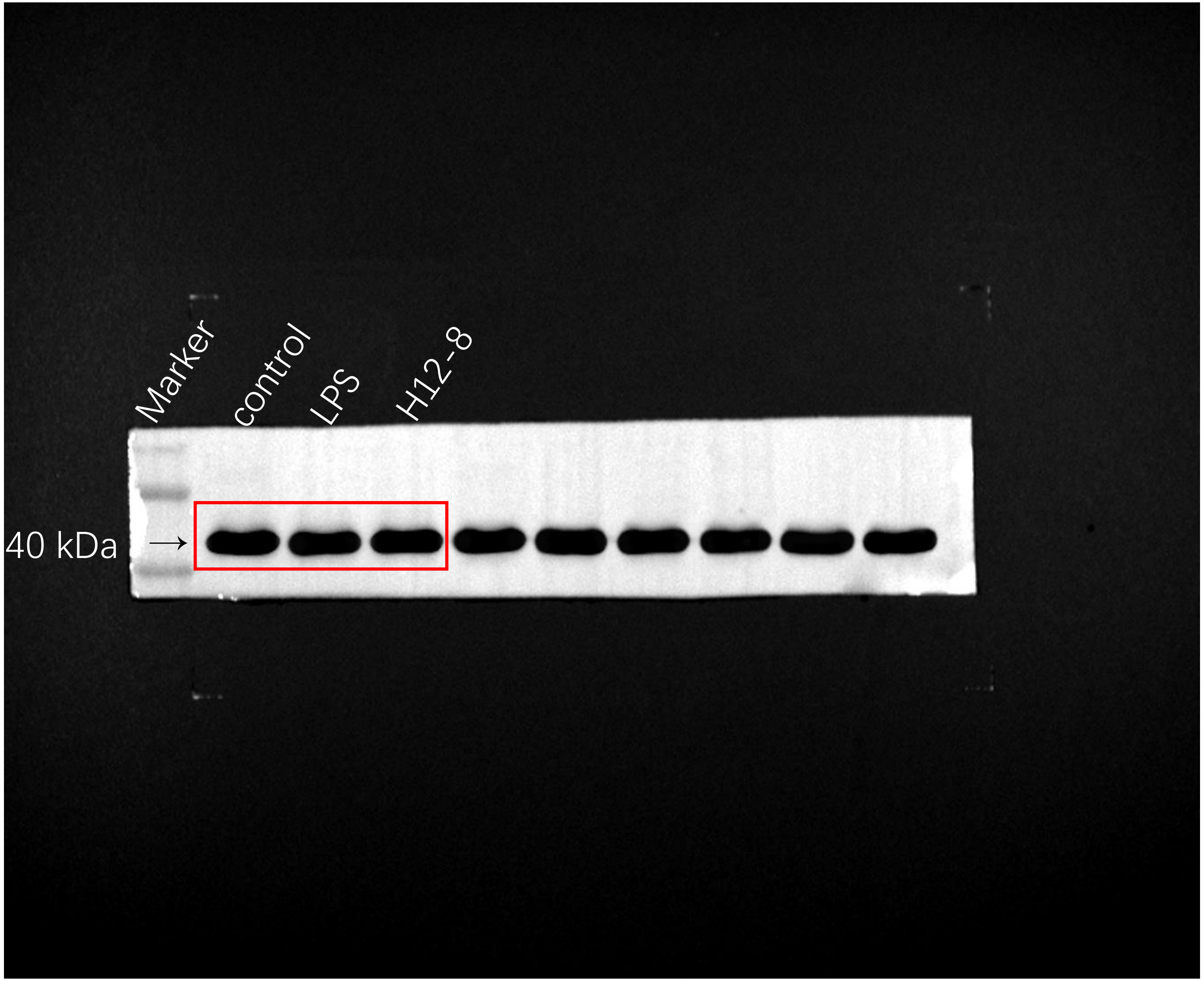


the cropped image in the manuscript were highlighted in red

Full unedited gel/blot of IL-1β for Figure 6D

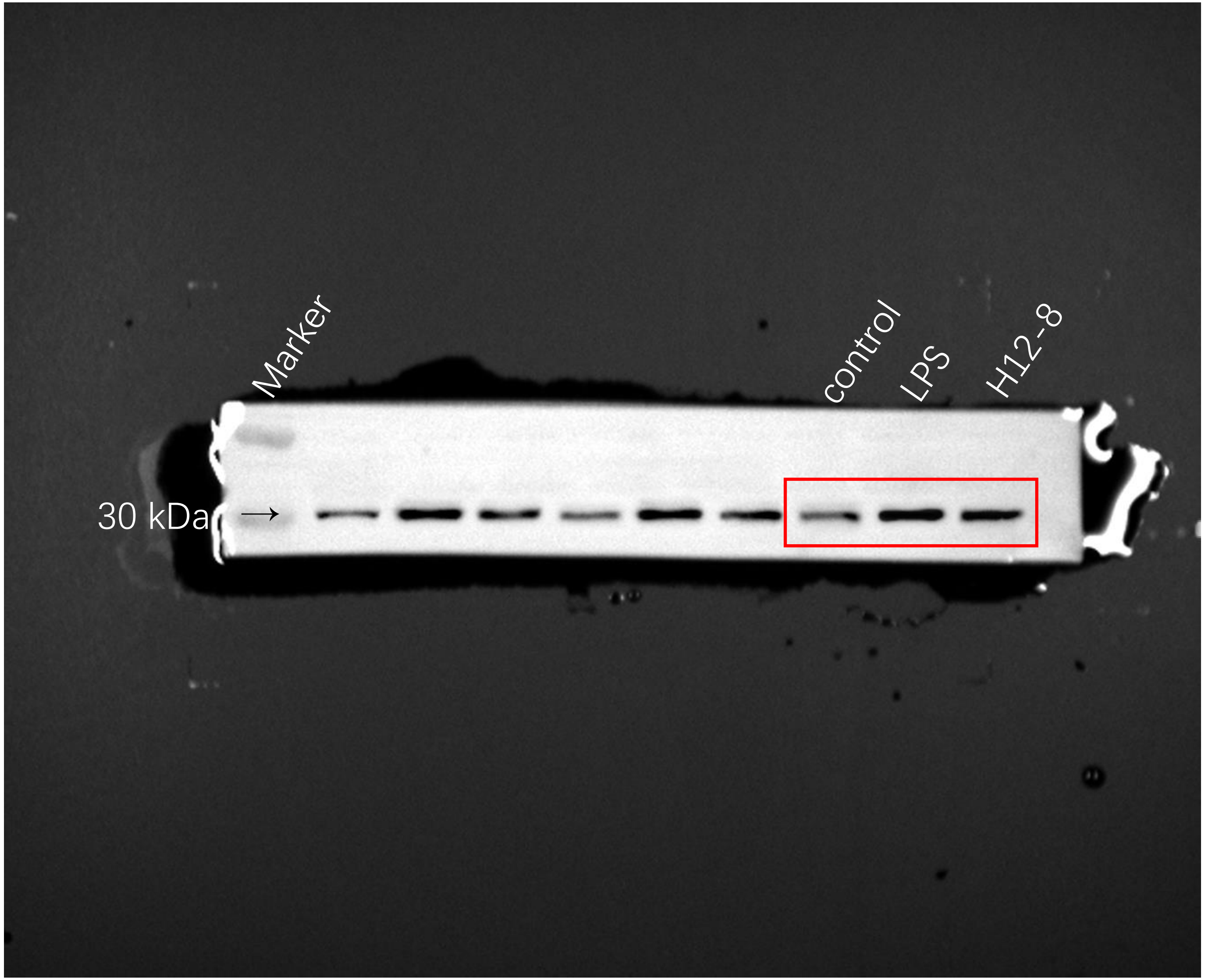


the cropped image in the manuscript were highlighted in red

Full unedited gel/blot of IL-6 for Figure 6D

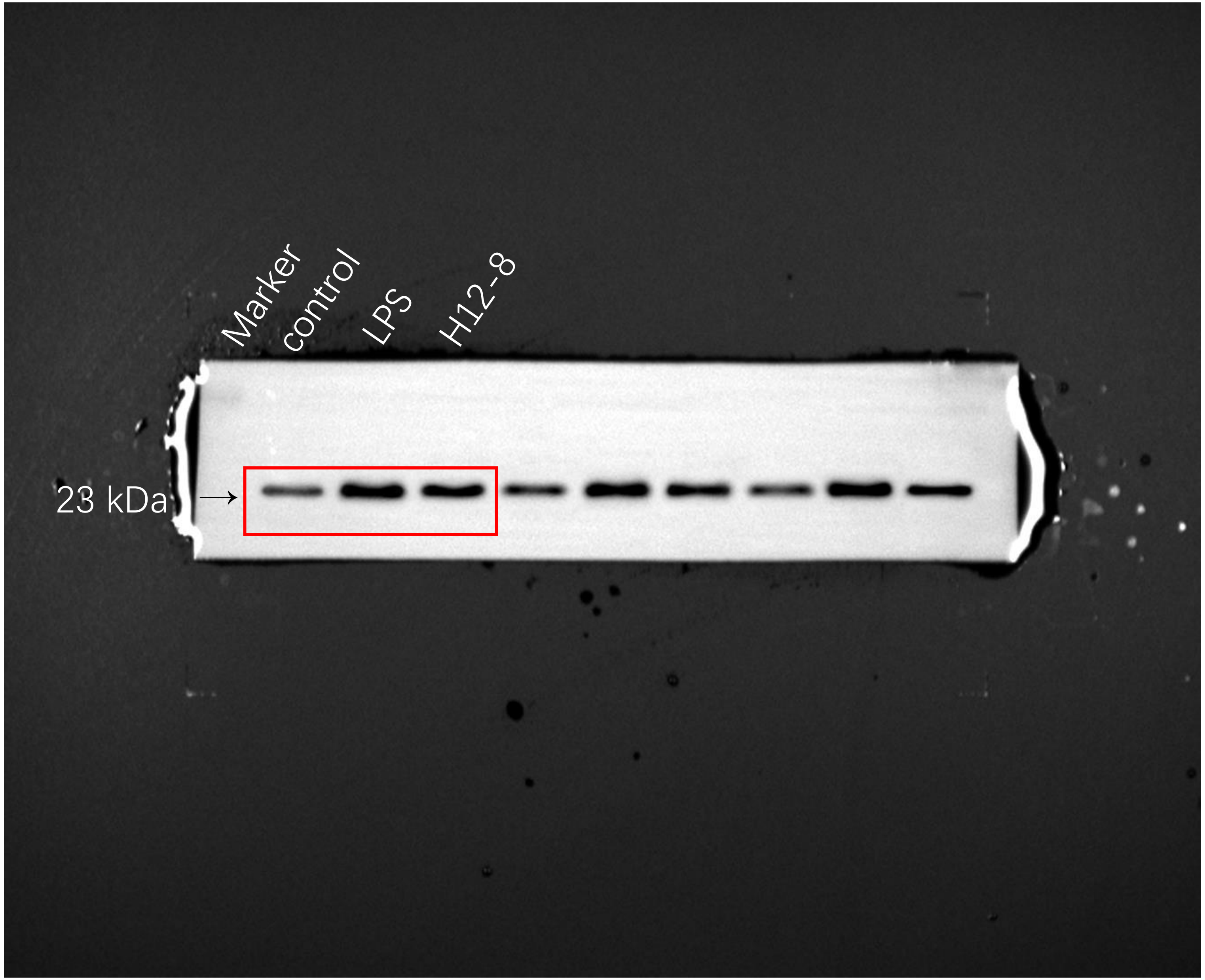


the cropped image in the manuscript were highlighted in red

Full unedited gel/blot of iNOS for Figure 6D

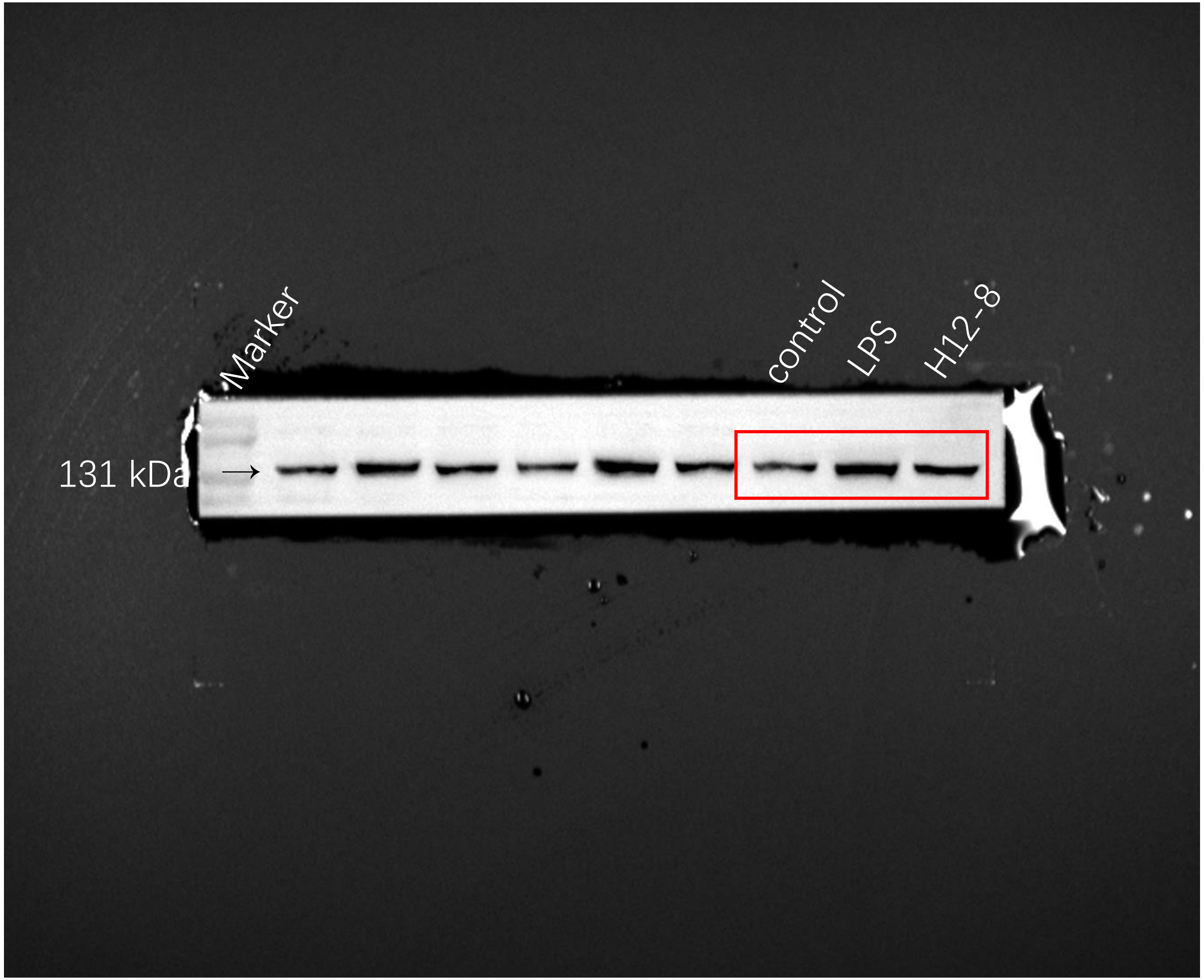


the cropped image in the manuscript were highlighted in red

Full unedited gel/blot of TNF-α for Figure 6D

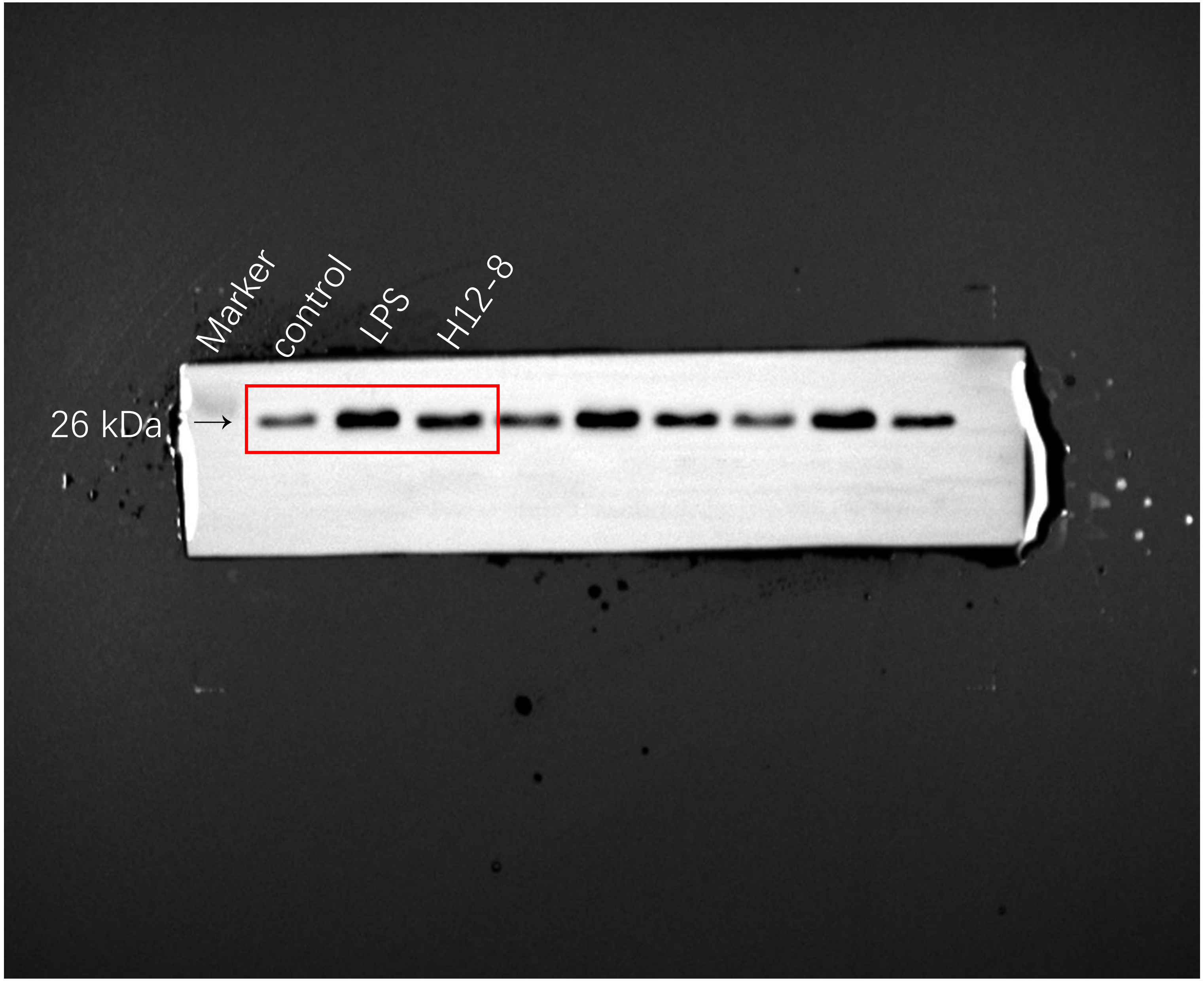


the cropped image in the manuscript were highlighted in red
